# A Balanced Risk–Benefit Analysis to Determine Human Risks Associated with Pyrrolizidine Alkaloids (PA)—The Case of Tea and Herbal Infusions

**DOI:** 10.3390/nu9070717

**Published:** 2017-07-07

**Authors:** Michael Habs, Karin Binder, Stefan Krauss, Karolina Müller, Brigitte Ernst, Luzia Valentini, Michael Koller

**Affiliations:** 1 Faculty of Medicine, LMU—University of Munich, 80333 Munich, Germany; info@michael-habs.de; 2 Didactics of Mathematics, University of Regensburg, 93053 Regensburg, Germany; Karin.Binder@mathematik.uni-regensburg.de (K.B.); stefan1.krauss@mathematik.uni-regensburg.de (S.K.); 3 Centre for Clinical Studies, University Hospital Regensburg, 93042 Regensburg, Germany; Karolina.Mueller@ukr.de; 4 General Medicine Unit, University Hospital Regensburg, 93053 Regensburg, Germany; dr.b.ernst@t-online.de; 5 Institute of Evidence-based Dietetics, University of Applied Sciences Neubrandenburg, 17033 Neubrandenburg, Germany; valentini@hs-nb.de

**Keywords:** pyrrolizidine alkaloids (PA), risk–benefit analysis, tea and herbal infusions

## Abstract

Humans are exposed to pyrrolizidine alkaloids (PA) through different sources, mainly from contaminated foodstuff. Teas and herbal infusions (T&HI) can be contaminated by PA producing weed. PA can possess toxic, mutagenic, genotoxic, and carcinogenic properties. Thus, possible health risks for the general population are under debate. There is a strong safety record for T&HI and additionally epidemiological evidence for the preventive effects of regular tea consumption on cardiovascular events and certain types of cancer. There is no epidemiological evidence, however, for human risks of regular low dose PA exposure. Recommended regulatory PA-threshold values are based on experimental data only, accepting big uncertainties. If a general risk exists through PA contaminated T&HI, it must be small compared to other frequently accepted risks of daily living and the proven health effects of T&HI. Decision making should be based on a balanced riskbenefit analysis. Based on analyses of the scientific data currently available, it is concluded that the benefits of drinking T&HI clearly outweigh the negligible health risk of possible PA contamination. At the same time, manufacturers must continue their efforts to secure good product quality and to be transparent on their measures of quality control and risk communication.

## 1. Introduction

Pyrrolizidine Alkaloids (PA) are synthesized by many plants and occur ubiquitously in the human environment. More than 660 PA in 6000 plants have been described [1,2,3]. They can contaminate biological products, including foodstuff, herbal remedies, supplements, and beverages [4,5].

The toxicity of PA only occurs after metabolic activation. Different competing metabolic pathways can lead to detoxification or poisoning. PA tested for toxicity differ broadly in experimental toxic potency [3,6]. Toxicity depends on chemical structure, is species-specific, modulated by gender, age, and the current metabolic situation [1]. Experimental toxicology shows PA to possess acute and chronic toxicity. The primary site for metabolic activation and the main target organ is the liver [7]. Hepatic occlusive disease, also called hepatic sinusoidal obstruction syndrome, is the typical result of acute PA poisoning, well documented experimentally and in livestock poisoning [8].

Some human anecdotal cases of PA associated liver damage have been described in the literature, of which only a few cases are well documented [2,6,9,10,11]. The lung can be affected by PA if pyrrolic metabolites escape from the liver and damage pulmonary arterioles [8]. Single case reports show transplacental exposure to lead to acute liver failure of the fetus without signs of maternal toxicity [12,13,14]. In developed countries one would expect primarily chronic damages resulting from long-term, low-dose exposure [2,6,9].

If teas (derived from *Camellia sinensis*, usually containing caffeine) and herbal infusions (derived from herbs and/or fruits, usually caffeine-free) (T&HI) are frequently contaminated, they always can become a major source for human exposure with the involved contaminants due to the worldwide extensive habit of drinking T&HI [15]. Correspondingly, T&HI are a prominent case to analyze the possible impact of PA contaminations in food. To assess the human safety of drinking T&HI, which can be contaminated with PA, numerous facts need to be considered. The traditional purpose of effective risk assessment and risk control is to prevent morbidity and mortality in humans.

However, regarding drinking T&HI, a balanced risk–benefit analysis is necessary that includes both, the potential risks of PA contaminations and the beneficial effects of drinking tea in general. The available evidence suggests that drinking T&HI is a beneficial custom resulting in improved health conditions [16,17,18].

The present paper provides a comprehensive overview of the current state of knowledge on PA and PA research, including evidence from experimental and human trials for health effects as well as toxicity, pathogenesis, and etiology of toxic effects as well as limitations of modern analytical detection methods. Finally, we try to put PA-related risks in perspective with more general health and everyday life risks. Taken together, this should enable stakeholders and consumers to engage in evidence-based, rational risk communication and informed decision making.

## 2. Health Effects and Safety of Consuming Tea and Herbal Infusions

Many beneficial health effects of drinking teas have been reported [16,17,19,20]. Recently tea in health and disease prevention has been reviewed [21]. In the last decade, more than 3000 studies, including fifty epidemiologic studies of the association between tea drinking and cancer risk reduction, have been published. A correlation between tea consumption and reduced cancer risk has been described for colon cancer, breast cancer, cancer of the ovary, and prostate cancer [22].

Epidemiological data from cohort studies and cross-sectional studies suggest a strong association between drinking green tea and a reduced incidence of liver diseases including hepatocellular carcinoma and liver cirrhosis [23,24]. A meta-analysis of observational studies on the association between tea drinking and a risk reduction for depression produced a dose response relationship: A linear association between tea consumption and risk decrease was shown with an increment of three cups/day leading to a risk decrease of 37% [25].

Moreover, tea consumption has been associated with a reduced risk to develop type 2 diabetes showing a linear inverse connection between tea drinking and type 2 diabetes [26]. With every additional two cups/day the risk to develop diabetes is reduced by 4.6% [27].

In addition, for cardiovascular diseases observational studies present an association between increasing tea consumption and risk reduction. A recent meta-analysis reported an increase in tea drinking by three cups/day being associated with a significantly reduced relative risk for coronary heart disease of 0.73, for cardiac death of 0.74, for stroke of 0.82, for cerebral infarction of 0.84, and for cerebral hemorrhage of 0.79 [28].

A Canadian qualitative study reported results from interviews with women who participated in the occupations of a Japanese tea ceremony [29]. Engaging in the tea ceremony enabled the sharing of common emotions, a sense of ongoing personal development, concentration, and a feeling of presence. The investigation shows that the culture of preparing and drinking tea can help to de-stress and to support living life mindfully, and to facilitate psychological well-being and mental balance beyond pharmacological effects [29]. The conscious habit of drinking tea is often rated as a desirable social behavior with positive connotations embedded in a traditional cultural context.

The polyphenols of *Camellia sinensis* with epigallocatechin-3-gallate as a lead constituent possess potent antioxidant capacity linked to the prevention of oxidative stress, modulation of toxicokinetic carcinogens and prevention of DNA adduct formation as possible modes of action [26]. There is also additional evidence that black tea drinking can improve recovery from psychophysiological stress [30].

In contrast to tea, popular herbal infusions (HI, herbal teas, and tisanes) used for medical purposes are made from, among others, chamomile, cinnamon, fennel, ginger, lemon balm, nettle, peppermint, rosemary, and valerian [10,11]. HI used as herbal medicinal products have medical indications either based on traditional use (Table 1) or supported by clinical studies leading to well-established indications. As medical products, they have a positive ris-benefit ratio as a prerequisite for market access. The herbals given in Table 1 are also used in common HI. No harmonized frame exists among the European countries what is regarded as food versus medical product. The Tea & Herbal Infusions Europe list of herbals considered as food is a compendium of the different herbal materials consumed as food ingredients in herbal teas and fruit teas in Europe [31]. It names several hundreds of plants and part of plants currently used by the herbal infusions trade. National use of HI is driven by tradition and behavioral trends. In 2013, the consumption of HI (including fruit teas) in Germany by far exceeded the use of tea (per capita consumption 40 L vs. 28 L). Leading single herb teas were peppermint leaf, fennel fruit, and chamomile flower [32].

In summary, there is consistent and increasing evidence based on experimental, clinical, and epidemiologic data that drinking T&HI is a healthy habit resulting in improved health conditions.

### Safety Assessment of Drinking Tea

Caffeine is the best documented bioactive ingredient in tea. When individuals consume moderate amounts, positive effects on human behavior become apparent through increased alertness and vigilance and reduced fatigue [40]. Reports on negative effects (increased anxiety, sleep disorders, and fine motor control affection) are linked to very large amounts given to sensitive groups (e.g., patients with anxiety disorders) [32]. The evidence clearly shows that levels consumed by most people have largely positive effects on behavior [40]. In a recent scientific opinion, the European Food Safety Authority (EFSA) Nutrition and Allergies (NDA) Panel concluded caffeine intakes from all sources up to 400 mg consumed throughout a day do not give rise to safety concerns for healthy adults in the general population [40]. In Europe, the mean daily caffeine consumption from tea and coffee is below this safety value in all 17 analyzed countries [40], however, in seven of these countries the 95% percentile estimate is above 400 mg [41], suggesting that at least 5% of the population consumes amounts above the safety threshold.

Three Cochrane Reviews dealing with green or black tea preparations have been published [19,20,42]. They studied green tea interventions for weight control, green and black tea for primary prevention of cardiovascular disease, and green tea for cancer prevention. Tea preparations with different standardization of active ingredients were pooled, and studies were performed in various patient groups and cultural settings. The controlled situation of interventional studies has the convincing advantage that possible unwanted effects will be far better identified than in daily use. That is why the results on safety from these three intervention reviews are of major importance. In the weight loss and weight maintenance review [42] is summarized that most adverse effects, such as nausea, constipation, abdominal discomfort, and increased blood pressure are mild to moderate and partly unrelated to green tea intervention. In the prevention studies of cardiovascular disease [19], the only adverse events measured were unlikely to be related to the intervention. From the cancer prevention trials the authors conclude that drinking green tea appears to be safe at moderate, regular, and habitual use [20].

Neither *Camellia sinensis* nor one of the common herbals used for making infusions was shown to produce PA. If PA occur in teas, it is the result of contamination with weeds.

We conclude that the habit of regularly drinking T&HI in usual amounts is safe. Therefore, we focus on the possible risk of PA contamination.

## 3. PA Toxicity: Analytical Detection, Pathogenesis, and Etiology

### 3.1. Analytical Detection of PA

Today, mass spectrometry (MS) can analyze PA at trace levels. In principle, two MS based approaches are applied, either in combination with gas chromatography (GC) or high performance liquid chromatography (HPLC) in tandem mass-spectrometry (MS/MS) mode [43,44].

A limited but increasing number of validated reference standards for PA are commercially available. The German Institute for Risk Assessment (BfR) recently listed 35 PA reference standards [2,9]. Due to structural diversity, low concentrations, and ubiquitously existence, PA analysis in the environment and in processed products poses substantial challenges. Validation of specific detection methods and strategies for screening processes are in progress. A current inter-laboratory comparison study for PA in animal feed using spiked and incurred material showed 3 out of 12 laboratories scored consequently positive or negative results. Two laboratories reported false positive results with a blank sample. Inter-laboratory variations led to the conclusion that the methods used for PA detection need further development for accurate estimation of contaminated feed [45]. Another inter-laboratory comparison organized by BfR with 31 participants investigated three PA contaminated herbal tea samples, one rooibos tea sample and a dissolved PA mixture. The observed discrepancies between the results of the participating laboratories showed room for methodical improvement and better standardization of operating procedures and performance criteria [46]. For comparability of analytical results, EMA Committee on Herbal Medicinal Products (HMPC) has requested that the European Pharmacopoeia considers development of an appropriate analytical method for PA a matter of priority [47]. EFSA and BfR recommend for sampling to use the Commission Regulation (EC) No 401/2006 laid down for mycotoxins as guidance for the control of the levels of PA in foodstuff [48].

Some health authorities have set a limit of 1.0 µg PA per day for the final product during a transitional phase. As a base for preventive measures the focus should be on the effects of combined human exposure of different PA from different sources, but no health-based guidance value has been established for the daily sum of PA from different sources. There are no binding test methods currently available. Recommendations from regulatory authorities prefer to measure the sum of quantified PA obtained by using validated reference substances of individual PA [2,5,9].

In summary, the threshold values discussed so far have the status of guidance, but not of legally binding limits. The same is true for analytical detection methods, which have not been harmonized yet [2,3,9].

### 3.2. PA Induced Toxicity: Pathogenesis

The chemical structure defines the toxicity of PA. Only 1,2-dehydropyrrolizidine-alkaloids are relevant for toxic effects [49]. PA without this structural feature are considered non-toxic. The native parent PA are nontoxic. Toxicity requires metabolic activation of highly reactive compounds.

Animal studies have shown that reactive metabolites are formed after metabolic activation via CYP 3A4 enzymes [42]. These alkylating reactive metabolites bind to macromolecules, including DNA.

Toxicodynamic investigations in mammals show quick absorption across the gastrointestinal tract, transport to the liver as the primary site for metabolic activation, renal excretion, and transplacental transport as well as transmission of water-soluble metabolites into the lung and skim breast milk [7]. Ingested PA are rapidly metabolized in the liver and the excretion of unchanged alkaloid and of most metabolites is rapid as well [6]. The European Medicines Agency concluded from the available toxicokinetic experimental data that within a few hours, only a relatively small proportion of the applied dose remains in the body, much of it in the form of metabolites bound to tissue constituents [4]. It is unlikely that a significant amount of unchanged alkaloid will remain in the body after the first day [4].

Metabolism steps involving cytochromes P-450 and flavin-containing monooxygenases can either lead to activation (toxification) or to detoxification [4].

The activation pathway is oxidation of the PA to form the dehydropyrrolizidine derivative (also referred to as the dehydronecine). Further biotransformation involves enzymatic or non-enzymatic glutathione conjugation or hydrolyses at the ester bond to form the dehydropyrrolizidine. Detoxification occurs by esterase cleavage leading to release of the necine base and necic acid(s). *N*-oxidation is generally catalyzed by a variety of mixed function oxidases, involving cytochromes P-450 and flavin-containing monooxygenases. *N*-oxides are highly water soluble and rapidly excreted via urine [1,4,6].

The pathogenesis of hepatotoxicity following PA exposure is only partially defined. Already for the hepatocyte, six different mechanisms of liver injury are known [49]. In PA liver toxicity, not only the hepatocytes are involved but also sinusoidal endothelial cells and central vein endothelial cells. Hepatocytes and sinusoidal endothelial cells metabolize the PA monocrotaline to toxic intermediates, but sinusoidal cells seem more sensitive to toxicity than hepatocytes. It has been discussed that the susceptibility of this cell type is due to a lower glutathione content leading to a greater intracellular exposure to toxic metabolite(s) due to a lower capacity for detoxification. This viewpoint is strengthened by the finding that in the monocrotaline model a concomitant infusion of glutathione dose dependently protected rats from development of hepatic veno-occlusive disease [50,51]. It is obvious that hepatic toxicity of monocrotaline is triggered by an overload of the detoxification systems of the liver. This finding suggests a threshold dose for this type of toxicity.

The toxicities of individual PA in biological systems are not only based on PA structural features but also on the pattern of expression and the selectivity of the CYP isoforms present [52]. Inter-individual enzyme concentrations of liver CYP3A4 and CYP3A5 vary over a 30-fold range [1,8]. Obviously individual susceptibility varies greatly within species and between species, gender, and individuals and over time, triggered by disposition and external factors, e.g., exposure to enzyme inducers. It remains unclear whether these differences are qualitative and/or quantitative in nature [53]. Gene expression profiling of liver genes of rats fed comfrey or dosed with the PA senecionine and riddelliine are an initial approach for revealing biological pathways and networks associated with toxicity and carcinogenicity induced by PA [54]. Genome transcriptome analysis in primary human hepatocytes exposed to four different PA, i.e., echimidine, heliotrine, senecione, and senkirkine showed all four PA to regulate a great number of genes in common, proposing similar molecular mechanisms, although the extent seems to differ [55].

Most primary liver tumors arise based on chronic liver inflammation subsequently inducing fibrogenesis and, ultimately, liver cirrhosis. The constant inflammatory cell death also promotes the development of phenotypic and molecular heterogeneity characteristic for hepatocellular carcinomas [56]. The presumption of similarity of rodents to humans may be test compound-specific [57] and the relevant mode of action for PA carcinogenesis in high doses in rodents may be different from the mode of action of low doses in humans.

### 3.3. PA Induced Toxicity: Etiology

No long-term animal studies have investigated the effects of food or beverages contaminated with PA. From long-term studies in rodents with different Ginkgo extracts it is known that toxic effects, including carcinogenicity, depend on the specific composition of the investigated extracts. Single components of an herbal extract may show carcinogenic properties, but the complex extract does not [58]. To extrapolate from high-dose animal studies with single, chemically defined PA to manufactured products occasionally contaminated with varying (comparatively low dose) concentrations is highly speculative. It is not known which concentration of individual PA or PA combinations or PA containing extracts or infusions result in which metabolism pattern leading to detoxification, acute, sub-acute, or chronic toxicity [3,4,7]. Rules for changes of enzyme patterns induced by repeated dosing or by combination with other xenobiotica have not been established [59].

In experimental animals, livestock poisoning by hay and plants containing PA and in human case reports (including transplacentally exposed infants) of accidental poisoning with PA containing herbal mixtures in high doses lead to acute liver toxicity with veno-occlusive disease, a specific form of damage to the microcirculation of the liver, also called hepatic sinusoidal obstruction syndrome.

Cases of illness have been described in the literature, but there are only a few well documented cases [2,9]. In the general population one would expect primarily chronic damage, i.e., carcinogenic, mutagenic, and teratogenic effects and human hepatic veno-occlusive disease [6]. Human single case reports show that transplacental exposure can lead to acute liver failure of the fetus without signs of maternal toxicity [14].

Exposure to a single oral dose of 160 mg/kg body weight of monocrotaline is used as a reproducible animal model for this type of hepatotoxicity [60]. However, the dose used corresponds in a human equivalent dose of 1.5 g for an adult [61], which by orders of magnitude exceeds human exposure [41,62].

Human hepatic veno-occlusive disease is the most prominent hepatic lesion associated with PA poisoning, but in general it is a very rare event. Liver diseases associated with PA-contaminated grain, have been reported for Pakistan, India, and Afghanistan [63]. Diagnosis is usually based on symptoms and on patients’ reports of having ingested substances associated with pyrrolizidine alkaloids. The latest outbreak in Afghanistan occurred in 2008. Sixty-seven cases of human hepatic veno-occlusive disease were identified in a case-control survey. A total of 28,443 individuals were surveyed, and 199 controls were matched with cases [63]. Consumption of bread was strongly associated with these cases. Thirty-two samples of flour were collected and analyzed for PA by means of liquid chromatography/mass-spectrometry. Median total concentration of PA in 12 samples taken from houses with liver disease cases was 5.6 mg/kg compared to 2.7 mg/kg in 20 control samples. The authors observed that identified cases were significantly more likely to report frequent consumption of bread [63]. Correspondingly, flour samples taken from case houses were found to be contaminated with high levels of PA stemming from contamination with seeds of Heliotrope or Crotalaria varieties [63]. In modern agricultural professional practice, this type of grain contamination is unlikely to occur because contamination with PA synthesizing plants in this type of farming is negligible [12].

The toxic effects of PA consumed in large doses over a short period manifest in humans as veno-occlusive disease, the occlusion of central venules is pathognomonic. BfR reported a case of an adult who had eaten plant material containing PA which caused severe liver function disease [2,9]. Rasenack and coworkers informed on the occurrence of human hepatic veno-occlusive disease in a preterm neonate who was delivered by caesarean section and died shortly afterwards [60]. Post- mortem examination confirmed the diagnosis and content of PA in the liver of the fetus. Analysis of an herbal mixture which was used for cooking in the family demonstrated high amounts of PA, establishing the casual relationship [14].

No general safety limits for acute toxicity in humans have been established from epidemiological or clinical data.

## 4. PA Toxicity: How Do Experimental Models and Findings in Humans Fit Together?

### 4.1. Combination of Animal and Human Toxicity Data Demonstrate the Relevance of Analogous Manifestation of Toxicity

The carcinogenicity of PA contaminated food has never been investigated in long-term animal studies. Chronic animal toxicity of single PA or extracts containing PA results in hepatic vein occlusions, mutagenic, teratogenic, and carcinogenic effects [6]. In rodents, the signal tumor of chronic PA poisoning is hepatic hemangiosarcoma [64,65]. It is concluded that inter-species qualitative comparability of metabolism and toxicity is feasible but huge quantitative differences are obvious. It is an open question whether the differences in metabolic cell response between rodents and humans should disqualify animal data for quantitative risk assessment in humans [53].

Risk assessment extrapolating animal findings to humans quantitatively implies major uncertainties. The reliability of animal data to predict outcome in humans has been critically reviewed by different working groups [66,67]. In general, comparisons between animal and human data show that findings in animals were not reliably replicated in humans. Heywood reported that animal experiments poorly predict adverse effects of pharmaceuticals and concluded “the best guess for the correlation of adverse reactions in man and animal toxicity data is somewhere between 5 and 25%” [66]. Olson et al. [67] analyzed the concordance of the toxicity of pharmaceuticals in humans and animals in a multinational pharmaceutical company survey compiling data from 12 companies with 150 compounds in clinical development. Non-rodent experiments predicted 63% of human organ toxicity, studies in rodents 43%, respectively. These discrepancies are thought to be due to differences in physiology and metabolism.

The value of animal use in the field of regulatory toxicology relies on a codified set of highly standardized acute, repeated dosing, and long-term animal studies, many of them developed in the 1960s. Their relevance has been scrutinized as more modern concepts became available to predict human outcome after exposure to xenobiotics [68,69,70]. However, if rare tumor types found in humans correspond to tumors in animal tested with the same suspected carcinogenic agent this is a reasonable empirical argument for causality. Identical organotropism and comparable tumor pathology have been demonstrated for about 50% of the chemicals, which are accepted by the World Health Organization (WHO) as carcinogenic to humans [71].

Hemangiosarcoma of unknown origin (“spontaneous tumors”) occur frequently in mice, are unusual in rats [46], and are well known in cats and special breeds of dogs [72]. Comparative gene expression profiling studies in Golden Retrievers suggest genetic background to mold occurrence, phenotype and biological behavior of sporadic hemangiosarcomas, at least in dogs [73]. These findings reveal that the risk of developing hemangiosarcoma could depend upon genetic disposition.

Carcinogens associated with liver carcinomas and hemangiosarcomas of the liver in humans include Alpha-emitters, vinyl chloride, and arsenic. Intravenous injection of thorium dioxide induced hepatocellular carcinomas in hamsters and liver carcinomas, intrahepatic bile-duct carcinomas, and hemangiosarcomas in rats [71]. Hepatic angiosarcomas can be reproducibly induced in rodents by exposure to vinyl chloride in the air [74].

Rodent liver angiosarcomas develop following exposure to DNA reactive, genotoxic chemicals as well as following chronic exposure to non-DNA reactive, non-genotoxic xenobiotics. Cohen et al. [53] have composed different modes of action for different mechanisms leading to hepatic angiosarcoma in rodents. Dysregulated angiogenesis can lead to local hypoxia followed by an overexpression of hypoxia-inducible factor 1-alpha (HIF) and vascular endothelial growth factor (VEGF), macrophage activation and thus locally increased interleukin-6 (IL-6) concentration. Both, VEGF and IL-6, can stimulate endothelial cell proliferation. The European Medicines Agency (EMA) statement [4] describes stimulation of cell proliferation following local hypoxia as a mode of action for hepatic angiosarcoma in rodents following exposure to PA. There are several mechanisms by which carcinogens cause cancer [75,76]. The mechanisms include DNA-methylation, histone methylation and acetylation, micro-RNA expression, receptor binding (aryl hydrocarbon, nuclear, peroxisome proliferator, and hormonal receptors), cytotoxicity, hormonal imbalance, chronic inflammation, oxidative stress, inhibition of apoptosis, disturbances in cell to cell communication, and induction of cell proliferation [76]. Dose thresholds have been shown experimentally for tumor induction by non-genotoxic tumor development. There is no proof that genotoxic compounds lead to carcinogenicity only through gene mutations. The hypothesis for genotoxic compounds assumes that one DNA mutation is sufficient to initiate a cancer cell. This hypothesis has not yet incorporated the better understanding of molecular mechanisms of DNA repair and apoptosis [77]. There appear to be significant tissue-specific and species-specific differences between the responses of endothelial cells to xenobiotics. It remains unclear whether these differences are qualitative and/or quantitative in nature. To ultimately address the question of interspecies comparisons a better understanding of biological similarities and differences between rodents and humans is needed.

Corresponding findings in (experimental) animals and humans strengthen the plausibility of a causative relationship and thereby move up the importance of the experimental findings in the hierarchy of evidence [62]. As shown in the paragraph on the etiology of PA induced toxicity experimental data, livestock poisoning and accidental human intoxications are comparable in acute and subacute toxicity. After PA poisoning many patients recover almost completely if the alkaloid intake is discontinued; cases of liver fibrosis and cirrhosis have been reported but no case of human liver malignancies has been linked to PA ingestion [13,62].

In regulatory toxicology, we assume threshold doses for most toxic effects. PA have the potential to produce several forms of toxicity. Acute intoxications are a result of overload of metabolic detoxification pathways and can result in secondary consequences such as hepatic occlusive disease. These effects assume a dose threshold below which no signs of toxicity occur. Due to metabolic detoxification of low doses no manifestation of acute and subacute liver toxicity is expected in the general population of developed countries with high agricultural standards [2,6]. This still leaves the question unanswered: what is the risk of possible low-dose, long-term exposure to PA by food contaminants?

Different approaches have been suggested to translate animal doses to human exposure risks. These calculations try to bridge speciesspecific differences, e.g., in genetic diversity, life expectancy, and basic metabolic pathways [78]. FDA has published a guidance document describing the use of standard specific factors that allow conversion of animal doses in (mg/kg) to human doses in (mg/kg) using the body surface area as the common denominator [79].

European regulators favor the margin of exposure approach to translate doses used in animal experiments to human exposure. Safety factors (in general of 10,000) are introduced to compensate for the knowledge gap in translating the benchmark dose lower confidence limit 10% (i.e., 95% confidence limit of the lowest dose showing a specific toxic effect in 10% of the exposed animals) in animals to the human situation [43].

EFSA has used the margin of exposure approach to translate the benchmark lower dose confidence limit for a 10% excess cancer risk of 70 µg/kg body weight per day for induction of liver hemangiosarcomas by lasiocarpine in male rats to a reference point for comparison with estimated dietary human exposure [43]. The quality of estimates in translating experimental findings to the human situation very much determines how efficiently the toxicity of PA in humans is controlled and prevented.

Since in carcinogenesis experiments PA induce hepatocellular carcinomas and hemangiosarcomas of the liver in the rat, it is worthwhile to look for a match with analogous malignancies in humans.

### 4.2. Epidemiological Evidence

In 2012, 782,000 new cases of liver cancer were diagnosed worldwide [80]. In the United States, 39,230 new cases of primary liver cancer (hepatocellular carcinoma (HCC) and intrahepatic cholangiocarcinoma (HCV)) are estimated for 2016 [81]. Other sources report about 21,000 men and 8000 women to newly develop primary liver cancer annually, while 27,170 people (18,280 men and 8890 women) are estimated to die of primary liver cancer in 2016 [81,82]. Thus, for calculation purposes, 25,000 deaths/year and twice as many men affected than women seem reasonable figures.

The overall rates of HCC tripled between 1975 and 2005. This increase is explained by the rise in hepatitis B and C virus carriers [83]. Major risk factors for HCC are chronic infection with hepatitis C and hepatitis B virus, excessive alcohol consumption, obesity, diabetes, metabolic syndrome, all can lead to liver cirrhosis. Smoking and eating foods with aflatoxin burden have also been associated with HCC [84,85,86]. HCV has been associated with several diseases of the biliary tract or liver, such as primary sclerosing cholangitis, Caroli disease, cholelithiasis, cholangitis, liver fluke, and inflammatory bowel disease.

No epidemiological data exist to associate chronic low dose PA exposure with human disorders [2,9,62]. The total number of primary liver cancer minus known attributable risks leaves only a low to very low risk for unknown causes. Theoretically, a fraction thereof could be associated with PA intake. The risk estimate attributable to hepatic infections accounts for well over 80% of all primary liver cancers. Thus, less than 20% are left for all other well established factors and for cancer of yet unexplained etiology, including a theoretical minority portion associated to chronic PA exposure) [82,87,88,89,90].

By subtracting the cases attributable to hepatitis (25,000 − >20,000 = <5000), a theoretical number of <5000 new cases/year can be calculated for the US population. Within these <5000 cases must be those due to known risk factors besides hepatitis B and C. The possible impact of PA contamination stays speculative, but is negligible compared to the impact of the sum of tumors probably attributable to already well-recognized risks. Thus far, no clinical association has been described between human cancer and exposure to PA. Based on the extensive reports on the outcome of human exposure available in the literature, Prakash, Pereira, Reilly, and Seawright [91] concluded that, while humans face the risk of veno-occlusive disease and childhood cirrhosis by PA poisoning, PAs are not carcinogenic to humans. 2500 annual cases linked to PA uptake seem clearly above a reasonable educated guess.

Human hepatic hemangiosarcoma (HHA) is a rare tumor [92,93], one source states HHA to account for only 2% of primary liver malignancies. Other authors estimated that only about 10 to 25 such cases occur each year in the United States [94,95]. Zochetti reported its frequency to be 2.5 cases every 10,000,000 persons [96]. For calculative purposes, an annual figure of 100 HHA for the US population seems a reasonable upper end estimate of the order of magnitude.

Established etiologic factors for HHA are thorium dioxide in angiography [97], exposure to vinyl chloride monomer at the work place [98], and ingested inorganic arsenic [99]. Anabolic-androgenic steroids have been associated, too [100]. No evidence of a relationship between environmental exposure to vinyl chloride monomer and angiosarcoma of the liver has been built [93]. An association with low dose, long-term PA exposure is without any epidemiological evidence [91]. The risk to experience HHA due to PA contaminated biologicals is negligible. If it does exist, it must be minimal compared to other largely accepted risks of daily living.

The overall comparison of experimental evidence, case reports and epidemiological evidence highlights that qualitative acute and subacute toxicity data correspond. High PA doses lead to a comparable specific liver pathology across species. However, regarding long term, low dose exposure no link has been found between the liver carcinogenicity seen in rat animal models and the human situation. Since liver hemangiosarcomas are signal tumors for human carcinogens, the missing accordance between rats and humans in PA tumorigenesis does not support the hypothesis that low dose PA contamination of food implies a considerable human cancer risk. In contrast, a solid data base exists demonstrating health protecting effects of T&HI.

While the PA-tumor link is unproven in humans, there exists a solid and consistent data base for the health-protecting effects of T&HI.

## 5. PA Exposure in Humans: Main Sources, Ways to Avoid It, and Competitive Risks

### 5.1. Main Sources of Human PA Exposure

In 2015, an EFSA supporting publication assessed the occurrence of PA in animal and plant derived products, teas, herbal infusions, and food supplements across different regions in Europe [5]. A total of 1105 samples (746 of animal origin and 359 of plant origin) were covered. Animal derived samples were mostly free of PA, which could partly be due to limits of the analytical method showing variable recoveries and matrix interferences with meat. Milk showed occasionally (in 6% of 182 samples) low levels of individual single PA. The analysis of the (herbal) tea samples revealed that a high proportion (91% of 166 samples) contained one or more PA. Rooibos tea showed the highest concentration of the sum of PA (mean concentration 7.99 µg/L) and chamomile tea the lowest (3.67 µg/L). Analyses of tea sampled in the retail market have shown contaminations with PA in black, green, rooibos, melissa, peppermint, chamomile, fennel, nettle, and mixed herbal tea [101].

In a recent systematic review of controlled trials, a liver related safety assessment of the intake of green tea extracts concluded that adverse events associated with the liver are expected to be rare [102]. In 2016, EFSA published a dietary exposure assessment to PA in the European population via the consumption of plant-derived food [103]. T&HI were by far the main contributors to total PA exposure. Using a most recent data set submitted by THIE, the European association representing the producers and traders of tea and herbal infusions, the estimated PA exposure was lower in comparison with the original data set. This downward trend could be the result of manufactures to better control PA contamination [103]. In conclusion, the habit of drinking (herbal) teas should be regarded as the major source of human PA exposure.

In the same investigation, very high PA levels were reported for certain food supplements derived from plant material of herbals known to synthesize PA. The authors underline the need to better understand the sources and routes of plant derived PA contamination which are still largely unknown but necessary to effectively reduce the contamination levels and the exposure of consumers [103]. Another well-known source of PA in dietary supplements are herbal plants used in traditional Chinese medicine [64].

### 5.2. PA Contamination and Ways to Avoid and Control It

In general, herbs known to produce PA should be banned from food and beverages. Educated herbalists know the plants they want to collect. The quality of certified wild collection of herbal plant material depends on the education of herbal picking, i.e., identification of the right plants and using the right techniques and times for harvesting. PA producing weeds are a specific challenge in large scale industrial farming and production of herbal products and teas.

Guidelines to achieve good agricultural and collecting practice by adequate quality assurance systems have been developed, e.g., by WHO [104] and EMA [47]. Especially if agricultural machines are used for weeding and harvesting, it is most important to avoid adulteration with weed.

Stale seed bed is a useful weed control technique but involves personal inspections of cultivated land area prior to seeding, during growth of herbs and before harvesting. To identify and destroy PA synthesizing plants is labor-intensive and jeopardizes the cost of goods.

The use of herbicides in agriculture for weed control is associated with environmental risks and against the expectations of most consumers of herbal infusions and teas. However, selective herbicides provide an efficient method of in-crop weed control and the agricultural industry takes broad advantage of this opportunity [105].

Field robots in precision agriculture have been prototyped and shown to be able to establish weed control by sensor technology, algorithms, and pattern recognition allowing to identify weed and get rid of it mechanically [106]. Based on this technology, it could become possible to improve weed control early in the value chain. Many raw herbal materials for the manufacturing of teas, infusions, and supplements are still wild-crafted either due to impracticality or low commercial demand to cultivate the species. In these cases, education is the key to avoid contamination of the harvest with PA containing plants. While zero tolerance towards PA in foodstuff and herbal products is not an option, minimization of human exposure is an option. In this respect, it is important to be informed about everyday risks in general and about health risks associated and not associated with PA.

### 5.3. The Need to Consider Competitive Risks

There are different approaches to deal with competitive risks: One way is to compare the impact of different modifiable risk factors on the manifestation of the disease under consideration.

Without doubt hepatitis B and C are the main causes for primary liver cancer. Consequently, public health authorities focus on these risks [107,108].

Another way to look at competitive risk is to analyze the impact of tradeoffs. With focus on PA contamination in foodstuff, one way to escape tea associated PA contamination would be avoiding tea consumption and turn to alternative beverages, such as soft drinks.

An impressing example for the need to analyze competitive risks can be drawn from the data of the (EPIC)-Norfolk study aimed to evaluate the association of different types of sugar-sweetened beverages (SSB) with incident type 2 diabetes and determine the effects of substituting non-SSB for SSB and the population-attributable fraction of type 2 diabetes due to total sweet beverages [109]. In adjusted Cox regression analyses, positive associations were shown for soft drinks, sweetened milk beverages and artificially sweetened beverages but not for sweetened tea or coffee or fruit juice. The analyses showed that each 5% of higher intake of energy (as a proportion of total daily energy intake) from total sweet beverages was associated with an 18% higher risk of diabetes. Substituting one serving/day of water or unsweetened tea/coffee for soft drinks and for sweetened-milk beverages reduced the incidence of diabetes by 14–25%. The authors concluded if sweet beverage consumers reduced intake to below 2% energy, 15% incident diabetes might be prevented. If avoidance of tea or herbal infusions would lead to an increased consumption of non-alcoholic beverages with a higher caloric burden, an increase in type 2 diabetes must be expected. The Center for Disease Control and Prevention estimated for the U.S. population the number of Americans with type 2 diabetes in 2012 to be 27.85 million cases (including 7.75 million undiagnosed). Thus, a rise of 18% translates to about 5 million (including 1.4 million undiagnosed) additional cases [110].

## 6. Balanced Risk–Benefit and Risk Communication

### 6.1. Objective versus Subjective Risks

Risk can be described as the product of the probability of occurrence and the amount of damage. The concept of risk is a result of the great concerns in modern societies about the dangers of life. Public health concerns often reflect perceptions following the media coverage of unfortunate events such as foreign substances in food, outbreaks of mad cow disease, avian influenza, and the political decision process on glyphosate [111]. Recent examples of pyrrolizidine alkaloids in honey and other foodstuffs add to this picture. These circumstances lead to public distrust and uncertainties about food safety. The diversity of conflicting opinions and special interests characterizes most public discussions about health issues involving production and use of nature-derived products.

There is a hierarchy in source quality. Pseudo-facts can easily masquerade facts. It is necessary to distinguish between claims with different levels of evidence. The more assumptions are needed to translate (e.g., analytical and experimental) data to the real human situation, the less evidence for plausibility exists.

Management of public safety and health is a tool to overcome the gap between expert views and public perception. The only thing that matters in effective risk assessment and applying risk controls is to prevent morbidity and mortality in humans. To invest limited resources cost-effectively, it is necessary to prioritize possible interventions. Risk assessment must provide state-of-the-art information about specific quantified health risks and help to prioritize relative health risks for decision makers.

Life is full of unforeseeable factors, and individual risks have only very limited predictability for individual outcome. Risk comparison, however, is helpful to make decisions now based on a prediction of the future, which by nature is uncertain. The level of effectiveness in reaching a desired outcome (as an example, escape a liver tumor by change of personal lifestyle) is a continuum from highly effective to ineffective or harmful. Risk comparison can frame the available information in an information environment, which seems familiar or, at least, facilitates a better understanding [112,113,114].

Table 2 provides a risk ladder based on the most recent death statistics in Germany [115], its taxonomy follows ICD 10 [116]. We use this data base to have a common source for the risk comparison. All causes of death represent the disease without death due to secondary diseases, e.g., death due to viral hepatitis ignores death through secondary diseases as liver cirrhosis and primary liver cancers. Obviously, liver diseases should be a major German health concern, leading to 4 times more annual deaths than car accidents. Hemangiosarcomas in general and HHA are not recorded in the annual death statistic of Germany. One more lesson that can be learned from the risk ladder is that two of the most prominent causes of death that are rarely dealt with in public discourse are household accidents (10,000 deaths per million) and age (11,000 per million in the age range 60–65).

If PA contaminants ingested in low doses over a long time pose a human cancer risk, it is plausible to analyze hepatic malignancies, because the liver is the most likely target organ [8]. No epidemiological data exist to show a causal relationship between PA uptake and human cancer. However, absence of evidence is not evidence of absence [117]. Although we cannot see any human tumor due to PA ingestion, this does not prove from a formal point of view that PA do not cause malignancies in humans.

To frame the potential risk, we can look at the different types of primary liver tumors, ask what order of magnitude seems to be explained by established risk factors. We then take the remaining tumors of unknown etiology as basis for an estimate of a risk which partly could be related to PA. To put this number into perspective we compare it to other morbidity or mortality risks. The possible PA contamination of products of daily use can only play a negligible role in human liver deaths.

If we assume that HHA, the corresponding signal tumor to rodent liver angiosarcomas, is linked to the intake of ubiquitously existing PA, it is obvious that such an event would be extremely rare, practically undetectable, and extremely small compared to other largely accepted risks of daily living.

The available evidence suggests that drinking tea is a healthy custom resulting in improved health conditions.

**Figure 1:**
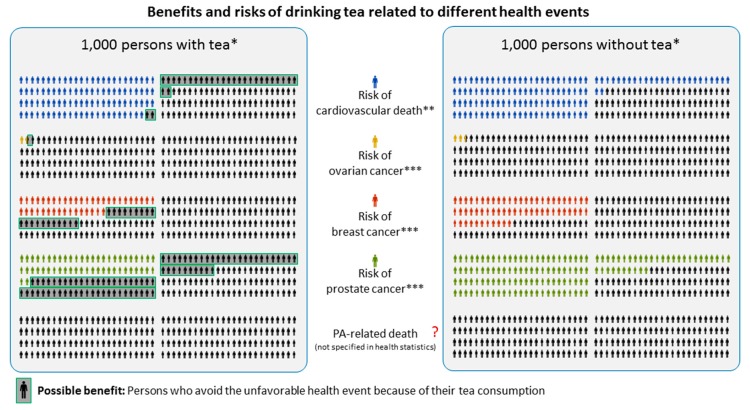
Icon array. Comparison of benefits and risks of drinking tea related to cardiovascular death [119], ovarian cancer [120], breast cancer [121], and prostate cancer [122]. All three cancer studies were case-control studies. Thus, reported data were adjusted to the lifetime risk of developing either ovarian (1:80), breast (1:7) or prostate cancer (1:6). * The precise definition of “with tea” and “without tea” differs in the considered studies, ** Among patients after myocardial infarction, *** Gender specific populations.

Based on our balanced risk–benefit analysis, abstinence from the consumption of T&HI is rated counterproductive since the positive effects of tea will be missed and alternative beverages may well be more hazardous, for example, sugar-sweetened soft drinks and alcoholic beverages. Nevertheless, one could argue that quitting tea consumption is an easy way of avoiding the hypothetical risk of PA intoxication. For a full picture of information, it is then useful to have a look at Figure 1. The icon arrays, based on ideas of the Harding Center for Risk Communication (Berlin, Germany), allow for comparing possible risks with benefits of drinking tea in selected health problems of 1000 persons under observation [118]. Health effects of tea consumption are particularly pronounced in the case of cardiovascular death. In a cohort study involving patients who experienced a myocardial infarction, 127 out of 1000 patients who consumed low amounts of tea died due to a cardiovascular event in the observation. In the group of tea consumers only 98 out of 1000 died. Thus, 29 out of 1000 patients profited from drinking tea. Comparable benefits could be derived from case-control studies involving patients with ovarian cancer, breast cancer, and prostate cancer. In contrast, PA-related health risks/benefits could not be quantified because of the lack of clinical or epidemiological data. These examples highlight the necessity to adopt a broad perspective when weighing risks and benefits. In the case of tea, avoiding a (hypothetical) risk may have the unexpected and unwanted consequence of rejecting a convincing health benefit.

### 6.2. PA Fact Collection to Provide Reference Points for Personal Decision Making

The present paper explores PA, tea consumption, and risk communication. The goal is to provide easy to understand information and thus empower the readers to balance their PA-related risk and the benefits of drinking T&HI to achieve an informed personal decision. Facts are compiled in four areas: occurrence of PA, biological activity (toxicity) of PA, PA risks and risk prevention, and the benefits of drinking teas.

Occurrence: PA are a group of chemically related products that are synthesized by many plants and occur in the human environment as contaminants. Modern analytical methods allow detecting even traces of PA in the human environment. The number of individually detectable PA is steadily increasing. PA have been detected recently in more and more foodstuff, including green, black, and herbal teas, honey, milk, lettuce, green vegetables as well as in herbal medicinal products and dietary supplements. No evidence exists for an increase in the overall human burden from PA during the last decades.

Biological activity and toxicity: PA become toxic after metabolic activation only, if doses exceed the detoxification capacity of the exposed organism. Conventional experimental toxicology studies show toxic effects with a specific form of hepatic veno-occlusive disease. In standardized tests, mutagenic, genotoxic, and tumor inducing properties, specifically hemangiosarcomas of the liver, have been described. Poisoning in ruminants by PA producing plants lead to damages comparable to findings observed in toxicity testing. Subacute liver toxicity, including hepatic veno-occlusive disease, was described repeatedly in people living in remote rural areas after contaminated flour was used in the bakery. Contamination resulted from adulteration with PA producing weeds. Acute poisoning in humans leading to toxic symptoms without delay is accidental and extremely rare. Single episodes have been reported after accidental consumption of food made from PA synthesizing plants.

PA risks and risk prevention: PA has attracted attention recently due to better analytical methods. For T&HI, a strong safety record in both healthy individuals and clinical populations is established. However, it is unknown if a human risk results from incorporating low doses of PAs on a regular basis. If this risk exists, no quantification of its health impact has been established. Prevention or reduction of human PA exposure aims to avoid possible harms resulting from long-term, low dose toxic burden. No tolerable human threshold doses for individual or the sum of PA have been established from real world evidence, and current recommended regulatory threshold values are based on experimental data only.

Quantitative extrapolation from PA animal data to humans must accept big knowledge gaps resulting in big uncertainties. To bridge these uncertainties, broad safety factors are applied to establish limit values which possess a reasonable certainty of doing no harm (Margin of exposure animal: human > 10,000). It is unknown if a general human risk through PA exists. T&HI are just one source of possible PA contamination through foodstuff, and this risk can only be partly responsible for the overall human risk due to PA. If a human risk for chronic toxicity of PA exists, it must be small in comparison to other, frequently accepted risks of human daily living. One way of reducing the risk of consuming PA is avoiding the consumption of T&HI. This would come at the expense of missing the positive and well-proven positive health effects of T&HI. There is no evidence that a healthier alternative exists by replacing the habit of drinking T&HI by switching to other beverages. There is significant evidence that replacing tea drinking by other beverages (e.g., high caloric soft drinks) could result in poorer health.

It is possible that higher PA risks exist for selected small groups under special circumstances, e.g., by transplacental exposure during pregnancy or in populations with underlying liver diseases resulting in compromised detoxification capacities.

Benefits of drinking teas: T&HI are worldwide consumed as tasty, healthy, and low-caloric beverages. Heating water before drinking reduces infectious risks, thus, in remote areas, drinking tea can prevent infections. Experimental studies, clinical studies and epidemiological data link tea consumption to beneficial health effects, including risk reduction for several types of cancer, type 2 diabetes, and cardiovascular diseases. Herbal teas are approved as herbal medicinal products by regulatory authorities. A positive risk–benefit ratio is mandatory for market access for all medicinal products. The evidence showing beneficial effects of T&HI is progressively increasing. However, no final scientific consensus exists on the extent of the health benefits resulting from daily drinking different types of T&HI. Despite the lack of convincing evidence from long-term intervention studies an increase in consumption of tea, with a negligible calorie load is encouraged.

## 7. Conclusions

Based on the current scientific data, drinking teas and herbal infusions is considered a recommendable habit associated with multiple health benefits.

At the same time, manufacturers must continue their efforts to secure good product quality, to keep PA contamination as low as possible and to be transparent on their measures of quality control and risk communication.

## Figures and Tables

**Table 1:** Herbal teas.

Herb	Indications	Evidence	Monograph
Chamomilla (*Matricaria chamomilla*)	digestive ailment, bloating, flatulence, restlessness, mild insomnia	anecdotal, traditional use	[33]
Cinnamon (*Cinnamomum verum*)	digestive ailment, mild diarrhea	anecdotal, traditional use	[34]
Fennel (*Foeniculum vulgare*)	dyspepsia, spasmodic ailments	traditional use	[11,35]
Ginger (*Zingiber officinale*)	dyspepsia, flatulence, prevention of nausea, vomiting, motion sickness	well established and traditional use	[36]
Lemon balm (*Melissa officinalis*)	mild symptoms of stress, anxiety and insomnia	traditional use	[37]
Nettle (*Urtica dioica*)	lower urinary tract symptoms related to benign prostatic hyperplasia	traditional use	[36]
Peppermint (*Mentha piperita*)	digestive disorders, nausea, abdominal pain	traditional use	[11]
Rosemary (*Rosmarinus officinalis*)	dyspepsia, mild gastrointestinal spasmodic disorders	traditional use	[38]
Valerian (*Valeriana officinalis*)	mild nervous tension and sleep disorders	traditional use	[39]

**Table 2:** Risk ladder.

Causes of Death Related to 1,000,000 Fatalities in Germany [108]	
**Cardiovascular disease**	385,000
**Cancer**	250,000
**Diabetes mellitus**	26,000
**Liver diseases**	16,000
**Age between 60 and 65**	11,000
**Household accidents**	10,000
**Liver cancer**	8500
**Car accidents**	3800
**Obesity**	2200
**Poisoning through pharmaceutical drugs**	2000
**Liver cancer (unexplained cause)**	1700
**Viral hepatitis**	1000
**Accidental poisoning**	750
**Unknown cause of death**	270
**Chronic hepatitis**	20
PA-related death *	?

* PA-related risks or deaths not listed.

## References

[1] Wiedenfeld H., Roeder E., Bourauel T., Edgar J. (2008). Pyrrolizidine Alkaloids. Structure and Toxicity.

[2] Bundesinstitut für Risikobewertung Frequently Asked Questions on Pyrrolizidine Alkaloids in Foods. http://www.bfr.bund.de/cm/349/frequently-asked-questions-on-pyrrolizidine-alkaloids-in-foods.pdf.

[3] Merz K.-H., Schrenk D. (2016). Interim relative potency factors for the toxicological risk assessment of pyrrolizidine alkaloids in food and herbal medicines. Toxicol. Lett..

[4] European Medicines Agency Public statement on the Use of Herbal Medicinal Products Containing Toxic, Unsaturated Pyrrolizidine Alkaloids (PAs). http://www.ema.europa.eu/docs/en_GB/document_library/Public_statement/2014/12/WC500179559.pdf.

[5] Mulder P.P., Sánchez P.L., These A., Preiss-Weigert A., Castellari M. (2015). Occurrence of Pyrrolizidine Alkaloids in food. EFS3.

[6] Wiedenfeld H. (2011). Plants containing pyrrolizidine alkaloids: Toxicity and problems. Food Addit. Contam. Part A.

[7] Habs M., Habs H., Forth W. (1991). Kanzerogene Naturprodukte: Risikobewertung pyrrolizidinhaltiger Arzneistoffe. Dtsch. Ärzteblatt.

[8] Wiedenfeld H., Edgar J. (2011). Toxicity of pyrrolizidine alkaloids to humans and ruminants. Phytochem. Rev..

[9] Bundesinstitut für Risikobewertung Vorläufige Empfehlungen des BfR zur Analytik von Pyrrolizidinalkaloiden (PA) in Kräutertee und Tee Analytspektrum und Probenahmeverfahren). http://www.bfr.bund.de/cm/343/vorlaeufige-empfehlungen-des-bfr-zur-analytik-von-pyrrolizidinalkaloiden-pa-in-kraeutertee-und-tee.pdf.

[10] European Medicines Agency Committee on Herbal Medicinal Products (HMPC). www.ema.europa.eu&gt;home&gt;committees&gt;HMPC&gt;Overview.

[11] Edwards S.E., Costa Rocha I.D., Williamson E.M., Heinrich M. (2015). Phytopharmacy. An Evidence-Based Guide to Herbal Medicinal Products.

[12] Wiedenfeld H. Aufnahmewege von PA Durch Direkte und Indirekte Intoxikation. https://www.ak-kreuzkraut.de/toxizität-mensch-tier/humangefährdung/aufnahmewege/.

[13] Pawar R.S. (2012). Pyrrolizidine alkaloids. Bad Bug Book: Foodborne Pathogenic Microorganisms and Natural Toxins.

[14] Rasenack R., Muller C., Kleinschmidt M., Rasenack J., Wiedenfeld H. (2003). Veno-occlusive disease in a fetus caused by pyrrolizidine alkaloids of food origin. Fetal Diagn. Ther..

[15] Statistical Portal Annual Per Capita Tea Consumption Worldwide as of 2016, by Leading Countries (In Pounds). https://www.statista.com/statistics/507950/global-per-capita-tea-consumption-by-country/.

[16] Khan N., Mukhtar H. (2013). Tea and health: Studies in humans. Curr. Pharm. Des..

[17] Serafini M., Rio D., N’Dri Y., Bettuzzi S., Peluso I., Wachtel-Galor S. (2011). Health Benefits of Tea. Herbal Medicine.

[18] Farzaneh V., Carvalho I.S. (2015). A review of the health benefit potentials of herbal plant infusions and their mechanism of actions. Ind. Crops Prod..

[19] Hartley L., Flowers N., Holmes J., Clarke A., Stranges S., Hooper L., Rees K. (2013). Green and black tea for the primary prevention of cardiovascular disease. Cochrane Database Syst. Rev..

[20] Boehm K., Borrelli F., Ernst E., Habacher G., Hung S.K., Milazzo S., Horneber M. (2009). Green tea (*Camellia sinensis*) for the prevention of cancer. Cochrane Database Syst. Rev..

[21] Chen Z.-M., Lin Z. (2015). Tea and human health: Biomedical functions of tea active components and current issues. J. Zhejiang Univ. Sci. B.

[22] National Cancer Institute Tea and Cancer Prevention. https://www.cancer.gov/about-cancer/causes-prevention/risk/diet/tea-fact-sheet.

[23] Jin X., Zheng R.-H., Li Y.-M. (2008). Green tea consumption and liver disease: A systematic review. Liver Int..

[24] Yin X., Yang J., Li T., Song L., Han T., Yang M., Liao H., He J., Zhong X. (2015). The effect of green tea intake on risk of liver disease: A meta analysis. Int. J. Clin. Exp. Med..

[25] Dong X., Yang C., Cao S., Gan Y., Sun H., Gong Y., Yang H., Yin X., Lu Z. (2015). Tea consumption and the risk of depression: A meta-analysis of observational studies. Aust. N. Z. J. Psychiatr..

[26] Yang C.S., Wang X., Lu G., Picinich S.C. (2009). Cancer prevention by tea: Animal studies, molecular mechanisms and human relevance. Nat. Rev. Cancer.

[27] Yang W.-S., Wang W.-Y., Fan W.-Y., Deng Q., Wang X. (2014). Tea consumption and risk of type 2 diabetes: A dose–response meta-analysis of cohort studies. Br. J. Nutr..

[28] Zhang C., Qin Y.-Y., Wei X., Yu F.-F., Zhou Y.-H., He J. (2015). Tea consumption and risk of cardiovascular outcomes and total mortality: A systematic review and meta-analysis of prospective observational studies. Eur. J. Epidemiol..

[29] Sakuae M., Reid D. (2012). Making Tea in Place: Experiences of Women Engaged in a Japanese Tea Ceremony. J. Occup. Sci..

[30] Steptoe A., Gibson E.L., Vuononvirta R., Williams E.D., Hamer M., Rycroft J.A., Erusalimsky J.D., Wardle J. (2007). The effects of tea on psychophysiological stress responsivity and post-stress recovery: A randomised double-blind trial. Psychopharmacology.

[31] Tea & Herbal Infusions Europe THIE Inventory List of Herbals Considered as Food. http://www.thie-online.eu/fileadmin/inhalte/Publications/HFI/2016/2016-06-24_PU_THIE_Inventory_List_of_Herbals_Considered_as_Food_final.pdf.

[32] Deutscher Teeverband e.V. Inlandskonsum auf Allzeithoch: Tee in Deutschland Beliebter denn je. http://www.teeverband.de/wirtschaft/pdf/2016-05-26_PM_WFT.pdf.

[33] European Medicines Agency, Committee on Herbal Medicinal Product European Union Herbal Monograph on *Matricaria Recutita* L., Flos. http://www.ema.europa.eu/docs/en_GB/document_library/Herbal_-_Herbal_monograph/2016/04/WC500204299.pdf.

[34] European Medicines Agency, Committee on Herbal Medicinal Product Community Herbal Monograph on *Cinnamomum Verum* J.S. Presl, Cortex. http://www.ema.europa.eu/docs/en_GB/document_library/Herbal_-_Community_herbal_monograph/2011/08/WC500110095.pdf.

[35] European Medicines Agency, Committee on Herbal Medicinal Product Community Herbal Monograph on Foeniculum Vulgare Miller Subsp. Vulgare Var. Vulgare, Fructus. http://www.ema.europa.eu/docs/en_GB/document_library/Herbal_-_Community_herbal_monograph/2009/12/WC500018464.pdf.

[36] European Medicines Agency, Committee on Herbal Medicinal Product Community Herbal Monograph on Zingiber Officinale Roscoe, Rhizoma. http://www.ema.europa.eu/docs/en_GB/document_library/Herbal_-_Community_herbal_monograph/2012/06/WC500128142.pdf.

[37] European Medicines Agency, Committee on Herbal Medicinal Product Community Herbal Monograph on *Melissa Officinalis* L., Folium. http://www.ema.europa.eu/docs/en_GB/document_library/Herbal_-_Community_herbal_monograph/2013/08/WC500147189.pdf.

[38] European Medicines Agency, Committee on Herbal Medicinal Product Community Herbal Monograph on *Rosmarinus Officinalis* L., Aetheroleum. http://www.ema.europa.eu/docs/en_GB/document_library/Herbal_-_Community_herbal_monograph/2011/01/WC500101493.pdf.

[39] European Medicines Agency, Committee on Herbal Medicinal Product European Union Herbal Monograph on *Valeriana Officinalis* L., Radix. http://www.ema.europa.eu/docs/en_GB/document_library/Herbal_-_Herbal_monograph/2016/04/WC500205376.pdf.

[40] Smith A. (2002). Effects of caffeine on human behavior. Food Chem. Toxicol..

[41] European Food Safety Authority (EFSA) Panel on Dietetic Products, Nutrition and Allergies (EFSA NDA Panel) (2015). Scientific Opinion on the safety of caffeine. EFSA J..

[42] Jurgens T.M., Whelan A.M., Killian L., Doucette S., Kirk S., Foy E. (2012). Green tea for weight loss and weight maintenance in overweight or obese adults. Cochrane Database Syst. Rev..

[43] European Food Safety Authority (EFSA) Panel on Contaminants in the Food Chain (CONTAM) (2011). Scientific Opinion on Pyrrolizidine alkaloids in food and feed. EFSA J..

[44] Crews C., Berthiller F., Krska R. (2010). Update on analytical methods for toxic pyrrolizidine alkaloids. Anal. Bioanal. Chem..

[45] Nijs M.D., Elbers I.J.W., Mulder P.P.J. (2014). Inter-laboratory comparison study for pyrrolizidine alkaloids in animal feed using spiked and incurred material. Food Addit. Contam. Part A.

[46] Bodi D., Pydde Y., Preiß-Weigert A. (2017). Internationale Laborvergleichsuntersuchung zur Bestimmung von Pyrrolizidinalkaloiden in Kräutertee und Rooibostee.

[47] Committee on Herbal Medicinal Product Guideline on Good Agricultural and Collecting Practice (GACP) for Starting Material of Herbal Origin. http://www.ema.europa.eu/docs/en_GB/document_library/Scientific_guideline/2009/09/WC500003362.pdf.

[48] The Commission of the European Communities COMMISSION REGULATION (EC) No. 401/2006: Of 23 February 2006 Laying Down the Methods of Sampling and Analysis for the Official Control of the Levels of Mycotoxins in Foodstuffs. http://eur-lex.europa.eu/LexUriServ/LexUriServ.do?uri=OJ:L:2006:070:0012:0034:EN:PDF.

[49] Lee W.M. (2003). Drug-induced hepatotoxicity. N. Engl. J. Med..

[50] DeLeve L.D., Ito Y., Bethea N.W., McCuskey M.K., Wang X., McCuskey R.S. (2003). Embolization by sinusoidal lining cells obstructs the microcirculation in rat sinusoidal obstruction syndrome. Am. J. Physiol. Gastrointest. Liver Physiol..

[51] Helmy A. (2006). Review article: Updates in the pathogenesis and therapy of hepatic sinusoidal obstruction syndrome. Aliment. Pharmacol. Ther..

[52] Ruan J., Yang M., Fu P., Ye Y., Lin G. (2014). Metabolic activation of pyrrolizidine alkaloids: Insights into the structural and enzymatic basis. Chem. Res. Toxicol..

[53] Cohen S.M., Storer R.D., Criswell K.A., Doerrer N.G., Dellarco V.L., Pegg D.G., Wojcinski Z.W., Malarkey D.E., Jacobs A.C., Klaunig J.E. (2009). Hemangiosarcoma in rodents: Mode-of-action evaluation and human relevance. Toxicol. Sci..

[54] Guo L., Mei N., Xia Q., Chen T., Chan P.-C., Fu P.P. (2010). Gene expression profiling as an initial approach for mechanistic studies of toxicity and tumorigenicity of herbal plants and herbal dietary supplements. J. Environ. Sci. Health Part C.

[55] Luckert C., Hessel S., Lenze D., Lampen A. (2015). Disturbance of gene expression in primary human hepatocytes by hepatotoxic pyrrolizidine alkaloids: A whole genome transcriptome analysis. Toxicology.

[56] El-Serag H.B. (2011). Hepatocellular carcinoma. N. Engl. J. Med..

[57] Holsapple M.P., Pitot H.C., Cohen S.M., Boobis A.R., Klaunig J.E., Pastoor T., Dellarco V.L., Dragan Y.P. (2006). Mode of action in relevance of rodent liver tumors to human cancer risk. Toxicol. Sci..

[58] Heinonen T., Gaus W. (2015). Cross matching observations on toxicological and clinical data for the assessment of tolerability and safety of Ginkgo biloba leaf extract. Toxicology.

[59] Lin G., Nnane I.P., Cheng T.-Y. (1999). The effects of pretreatment with glycyrrhizin and glycyrrhetinic acid on the retrorsine-induced hepatotoxicity in rats. Toxicon.

[60] DeLeve L.D., McCuskey R.S., Wang X., Hu L., McCuskey M.K., Epstein R.B., Kanel G.C. (1999). Characterization of a reproducible rat model of hepatic veno-occlusive disease. Hepatology.

[61] Reagan-Shaw S., Nihal M., Ahmad N. (2008). Dose translation from animal to human studies revisited. FASEB J..

[62] European Medicines Agency Public Statement on Contamination of Herbal Medicinal Products/traditional Herbal Medicinal Products with Pyrrolizidine Alkaloids: Transitional Recommendations for Risk Managementand Quality Control. http://www.ema.europa.eu/docs/en_GB/document_library/Public_statement/2016/06/WC500208195.pdf.

[63] Kakar F., Akbarian Z., Leslie T., Mustafa M.L., Watson J., van Egmond H.P., Omar M.F., Mofleh J. (2010). An outbreak of hepatic veno-occlusive disease in Western Afghanistan associated with exposure to wheat flour contaminated with pyrrolizidine alkaloids. J. Toxicol..

[64] Fu P.P., Yang Y.-C., Xia Q., Chou M.W., Cui Y.Y., Lin G. (2002). Pyrrolizidine Alkaloids: Tumorigenic Components in Chinese Herbal Medicines and Dietary Supplements. J. Food Drug Anal..

[65] Habs H., Habs M., Marquardt H., Roder E., Schmahl D., Wiedenfeld H. (1982). Carcinogenic and mutagenic activity of an alkaloidal extract of *Senecio nemorensis* ssp. fuchsii. Arzneim. Forsch..

[66] Heywwod R. Clinical toxicity—Could it have been predicted? Post-marketing experience. Proceedings of the Animal Toxicity Studies: Their Relevance for Man.

[67] Olson H., Betton G., Robinson D., Thomas K., Monro A., Kolaja G., Lilly P., Sanders J., Sipes G., Bracken W. (2000). Concordance of the toxicity of pharmaceuticals in humans and in animals. Regul. Toxicol. Pharmacol..

[68] National Research Council (2004). Intentional Human Dosing Studies for EPA Regulatory Purposes. Scientific and Ethical Issues.

[69] Ferdowsian H.R., Beck N. (2011). Ethical and scientific considerations regarding animal testing and research. PLoS ONE.

[70] Akhtar A. (2015). The flaws and human harms of animal experimentation. Camb. Q. Healthc. Ethics.

[71] International Agency for Research on Cancer (2012). IARC Monographs on the Evaluation of Carcinogenic Risks to Humans: Radiation.

[72] Schultheiss P.C. (2004). A retrospective study of visceral and nonvisceral hemangiosarcoma and hemangiomas in domestic animals. J. Vet. Diagn. Investig..

[73] Tamburini B.A., Trapp S., Phang T.L., Schappa J.T., Hunter L.E., Modiano J.F. (2009). Gene expression profiles of sporadic canine hemangiosarcoma are uniquely associated with breed. PLoS ONE.

[74] Hong C.B., Winston J.M., Lee C.C. (1980). Hepatic angiosarcoma: Animal model: Angiosarcoma of rats and mice induced by vinyl chloride. Am. J. Pathol..

[75] Adler S., Basketter D., Creton S., Pelkonen O., van Benthem J., Zuang V., Andersen K.E., Angers-Loustau A., Aptula A., Bal-Price A. (2011). Alternative (non-animal) methods for cosmetics testing: Current status and future prospects-2010. Arch. Toxicol..

[76] Basketter D. (2012). A roadmap for the development of alternative (non-animal) methods for systemic toxicity testing. ALTEX.

[77] Sancar A., Lindsey-Boltz L.A., Unsal-Kacmaz K., Linn S. (2004). Molecular mechanisms of mammalian DNA repair and the DNA damage checkpoints. Annu. Rev. Biochem..

[78] Nair A., Jacob S. (2016). A simple practice guide for dose conversion between animals and human. J. Basic Clin. Pharm..

[79] Food and Drug Administration Guidance for Industry: Estimating the Maximum Safe Starting Dose in Initial Clinical Trials for Therapeutics in Adult Healthy Volunteers. https://www.fda.gov/downloads/drugs/guidances/ucm078932.pdf.

[80] World Cancer Research Fund International. Cancer Facts & Figures: Worldwide Data.

[81] American Cancer Society (2016). Cancer Facts & Figures 2016.

[82] Centers for Disease Control and Prevention Liver Cancer. https://www.cdc.gov/cancer/liver/.

[83] Altekruse S.F., McGlynn K.A., Reichman M.E. (2009). Hepatocellular carcinoma incidence, mortality, and survival trends in the United States from 1975 to 2005. J. Clin. Oncol..

[84] Liu Y., Wu F. (2010). Global burden of aflatoxin-induced hepatocellular carcinoma: A risk assessment. Environ. Health Perspect..

[85] Koh W.-P., Robien K., Wang R., Govindarajan S., Yuan J.-M., Yu M.C. (2011). Smoking as an independent risk factor for hepatocellular carcinoma: The Singapore Chinese Health Study. Br. J. Cancer.

[86] Wambui J.M., Karuri E.G., Ojiambo J.A., Njage P.M.K. (2017). Application of Probabilistic Modeling to Quantify the Reduction Levels of Hepatocellular Carcinoma Risk Attributable to Chronic Aflatoxins Exposure. Nutr. Cancer.

[87] Balogh J., Victor D., Asham E.H., Burroughs S.G., Boktour M., Saharia A., Li X., Ghobrial R.M., Monsour H.P. (2016). Hepatocellular carcinoma: A review. J. Hepatocell. Carcinoma.

[88] Bosch F.X., Ribes J., Diaz M., Cleries R. (2004). Primary liver cancer: Worldwide incidence and trends. Gastroenterology.

[89] Welzel T.M., Graubard B.I., Zeuzem S., El-Serag H.B., Davila J.A., McGlynn K.A. (2011). Metabolic syndrome increases the risk of primary liver cancer in the United States: A study in the SEER-Medicare database. Hepatology.

[90] Ananthakrishnan A., Gogineni V., Saeian K. (2006). Epidemiology of primary and secondary liver cancers. Semin. Interv. Radiol..

[91] Prakash A.S., Pereira T.N., Reilly P.E., Seawright A.A. (1999). Pyrrolizidine alkaloids in human diet. Mutat. Res..

[92] Falk H., Herbert J., Crowley S., Ishak K.G., Thomas L.B., Popper H., Caldwell G.G. (1981). Epidemiology of hepatic angiosarcoma in the United States: 1964–1974. Environ. Health Perspect..

[93] Huang N.-C., Wann S.-R., Chang H.-T., Lin S.-L., Wang J.-S., Guo H.-R. (2011). Arsenic, vinyl chloride, viral hepatitis, and hepatic angiosarcoma: A hospital-based study and review of literature in Taiwan. BMC Gastroenterol..

[94] Centers for Disease Control and Prevention (1997). Epidemiologic notes and reports: Angiosarcoma of the liver among polyvinyl chloride workers—Kentucky. Morb. Mortal. Wkly. Rep..

[95] Mani H., van Thiel D.H. (2001). Mesenchymal tumors of the liver. Clin. Liver Dis..

[96] Zocchetti C. (2001). Angiosarcoma del fegato nell'uomo: Considerazioni epidemiologiche. La Medicina del lavoro.

[97] Van Kampen R.J.W., Erdkamp F.L.G., Peters F.P.J. (2007). Thorium dioxide-related haemangiosarcoma of the liver. Neth. J. Med..

[98] International Agency for Research on Cancer (2012). IARC Monographs on the Evaluation of Carcinogenic Risks to Humans: Chemical Agents and Related Occupations.

[99] Chiou H.Y., Hsueh Y.M., Liaw K.F., Horng S.F., Chiang M.H., Pu Y.S., Lin J.S., Huang C.H., Chen C.J. (1995). Incidence of internal cancers and ingested inorganic arsenic: A seven-year follow-up study in Taiwan. Cancer Res..

[100] Falk H., Thomas L.B., Popper H., Ishak K.G. (1979). Hepatic angiosarcoma associated with androgenic-anabolic steroids. Lancet.

[101] Bodi D., Ronczka S., Gottschalk C., Behr N., Skibba A., Wagner M., Lahrssen-Wiederholt M., Preiss-Weigert A., These A. (2014). Determination of pyrrolizidine alkaloids in tea, herbal drugs and honey. Food Addit. Contam. Part A.

[102] Isomura T., Suzuki S., Origasa H., Hosono A., Suzuki M., Sawada T., Terao S., Muto Y., Koga T. (2016). Liver-related safety assessment of green tea extracts in humans: A systematic review of randomized controlled trials. Eur. J. Clin. Nutr..

[103] European Food Safety Authority (EFSA) (2016). Dietary exposure assessment to pyrrolizidine alkaloids in the European population. EFSA J..

[104] World Health Organization WHO Guidelines on Good Agricultural and Collection Practices (GACP) for Medicinal Plants. http://apps.who.int/iris/bitstream/10665/42783/1/9241546271.pdf.

[105] Green J.M. (2012). The benefits of herbicide-resistant crops. Pest Manag. Sci..

[106] Biber P., Weiss U., Dorna M., Albert A. Navigation System of the Autonomous Agricultural Robot “BoniRob”. Proceedings of the Workshop on Agricultural Robotics: Enabling Safe, Efficient, and Affordable Robots for Food Production.

[107] Perz J.F., Armstrong G.L., Farrington L.A., Hutin Y.J.F., Bell B.P. (2006). The contributions of hepatitis B virus and hepatitis C virus infections to cirrhosis and primary liver cancer worldwide. J. Hepatol..

[108] Centers for Disease Control and Prevention Viral Hepatitis. http://www.cdc.gov/hepatitis.

[109] O’Connor L., Imamura F., Lentjes M.A.H., Khaw K.-T., Wareham N.J., Forouhi N.G. (2015). Prospective associations and population impact of sweet beverage intake and type 2 diabetes, and effects of substitutions with alternative beverages. Diabetologia.

[110] Centers for Disease Control and Prevention (2014). National Diabetes Statistics Report, 2014: Estimates of Diabetes and Its Burden in the United States.

[111] Myers J.P., Antoniou M.N., Blumberg B., Carroll L., Colborn T., Everett L.G., Hansen M., Landrigan P.J., Lanphear B.P., Mesnage R. (2016). Concerns over use of glyphosate-based herbicides and risks associated with exposures: A consensus statement. Environ. Health.

[112] Gigerenzer G. (2003). Why does framing influence judgment?. J. Gen. Intern. Med..

[113] Spiegelhalter D.J. (2008). Understanding uncertainty. Ann. Fam. Med..

[114] Spiegelhalter D., Pearson M., Short I. (2011). Visualizing uncertainty about the future. Science.

[115] Statistisches Bundesamt (Destatis) Gesundheit: Todesursachen in Deutschland 2015. https://www.destatis.de/DE/Publikationen/Thematisch/Gesundheit/Todesursachen/Todesursachen.html.

[116] World Health Organization (2011). International Statistical Classification of Diseases and Related Health Problems.

[117] Altman D.G., Bland J.M. (1995). Statistics notes: Absence of evidence is not evidence of absence. BMJ.

[118] McDowell M., Rebitschek F.G., Gigerenzer G., Wegwarth O. (2016). A Simple Tool for Communicating the Benefits and Harms of Health Interventions. MDM Policy Pract..

[119] Mukamal K.J., Maclure M., Muller J.E., Sherwood J.B., Mittleman M.A. (2002). Tea consumption and mortality after acute myocardial infarction. Circulation.

[120] Larsson S.C., Wolk A. (2005). Tea consumption and ovarian cancer risk in a population-based cohort. Arch. Intern. Med..

[121] Kumar N., Titus-Ernstoff L., Newcomb P.A., Trentham-Dietz A., Anic G., Egan K.M. (2009). Tea consumption and risk of breast cancer. Cancer Epidemiol. Biomark. Prev..

[122] Le J., Xie L.P., Lee A.H., Binns C.W. (2004). Protective effect of green tea against prostate cancer: A case-control study in southeast China. Int. J. Cancer.

